# Long term health effects of NEET experiences: evidence from a longitudinal analysis of young people in Scotland

**DOI:** 10.23889/ijpds.v1i1.327

**Published:** 2017-04-19

**Authors:** Zhiqiang Feng, Kevin Ralston, Dawn Everington, Chris Dibben

**Affiliations:** 1 University of Edinburgh

## Background

This paper examines whether experiences of young people who are not in employment, education or training (NEET) are associated with adverse long-term outcomes in health. We used the Scottish Longitudinal Study (SLS), which includes information from the 1991, 2001, and 2011 censuses as well as from vital events, for a 5.3% representative sample of the Scottish population. Linked health data such as hospital admissions and prescribing in general practice are also available. We followed around 14,000 young people who were aged 16-19 in 1991 up to 2011.

## Method

We explored whether NEET young people in 1991 displayed higher risks of poor physical and mental health in the follow-up period. Poor physical health is measured by any admission into hospital and poor mental health is measured by prescription of anti-depressant and anti-anxiety medicine. We used descriptive and modelling approaches in our analysis. Covariates include a number of individual socioeconomic characteristics and local area characteristics in the models.

## Results

Our research found that over 40% of the cohort members have been admitted into hospital, while over 30% have been prescribed with anti-depressant and anti-anxiety drugs. The NEET status in 1991 appears to be associated with hospitalisation with adjusted odds ratio (OR) of 1.24 (95% Confidence Intervals (CIs): 1.08 – 1.42). Also the NEET experiences are associated with poor mental health with OR of 1.47 (95% CI: 1.27 – 1.71). Policy intervention is necessary in assisting NEET young people to re-engage in education or employment.

